# Cardiovascular disease in women: A review of spontaneous coronary artery dissection

**DOI:** 10.1097/MD.0000000000030433

**Published:** 2022-09-23

**Authors:** Bashar Khiatah, Sam Jazayeri, Naofumi Yamamoto, Tristen Burt, Amanda Frugoli, Dennis L Brooks

**Affiliations:** a Community Memorial Hospital, Internal Medicine Department, Ventura, CA, USA; b College of Osteopathic Medicine of the Pacific, Western University, Pomona, CA, USA; c Cardiology Associates Medical Group, Ventura, CA, USA.

**Keywords:** SCAD, CAD, ACS

## Abstract

Research has demonstrated the disproportionate quality of care for women with cardiovascular disease. These findings have prompted a renewed focus on cardiovascular disease awareness and disease prevention in women. Spontaneous coronary artery dissection (SCAD) is a significant cause of myocardial infarction (MI) and sudden death that primarily affects women. ongoing research has led to improved diagnostic capabilities and changes in approaches to initial and long-term management most importantly this research has provided evidence that SCAD is more common than previously thought and must be evaluated and treated differently from atherosclerotic MI. The difference between SCAD and atherosclerotic MI is highlighted in high rates of recurrent disease, gender distribution, association with exogenous hormones, pregnancy, migraine, physical and emotional stress triggers, concurrent systemic arteriopathies, and connective tissue disease. In this review, we provide updated insights and a summary of the epidemiology, risk factors, clinical presentation, diagnosis, treatment options, prognosis, and recurrence prevention of SCAD. We aim to provide a review of SCAD as a focus on cardiovascular disease awareness and disease prevention in women.

**Key points**

- The understanding of SCAD has been increasing with the development of more diagnostics and more research to assess the risk factors which are described here in this paper

Medical management of SCAD patients is discussed here in detail with the most recent update according to the current cardiologist consent

## 1. Introduction

Spontaneous coronary artery dissection (SCAD) is a significant cause of myocardial infarction (MI) and sudden death, specifically among young adults and women.^[1]^ It is defined as a spontaneous, non-traumatic, non-iatrogenic rupture of the coronary artery wall forming a false lumen and an intramural hematoma (IMH). This IMH under certain circumstances is capable of compressing the true artery lumen resulting in acute coronary syndrome (ACS) with myocardial ischemia.^[2]^ SCAD was first described in 1931 during the autopsy of a 42-year-old lady who had a violent retching attack before she dropped dead.^[3]^ For the next couple of decades, most of the evidence was derived from isolated case reports and small series of patients, resulting in a slow increase in disease pathophysiology^[4]^ Later on, more cases have been diagnosed with the incremental development of invasive diagnostic and therapeutic approaches.^[5]^ Since then, it has been shown that SCAD is more common and challenging than initially suspected. SCAD is often categorized depending on the predisposing condition that has been generally divided into atherosclerotic and nonatherosclerotic.^[6]^

## 2. Prevalence

About 4% of patients presenting with ACS have evidence of SCAD on coronary angiography. A large majority of patients with SCAD reaching up to 90% are females.^[7]^ In women under 60 years of age, SCAD comprises over one-third of ACS cases.^[8]^ It is no coincidence that the first described case of SCAD was a young female. It is possible that some of the poor outcomes identified in women with ACS could be from the complex diagnostic and treatment algorithms for SCAD. We hypothesize that SCAD is both misdiagnosed and underdiagnosed resulting in subtherapeutic treatments. The true prevalence and incidence rate across the general population remains unknown. However, SCAD recognition as a cause of ACS is increasing due to physicians’ awareness and increases in technology available to establish the diagnosis of SCAD.^[9]^

## 3. Physiopathology

**Figure 1.**
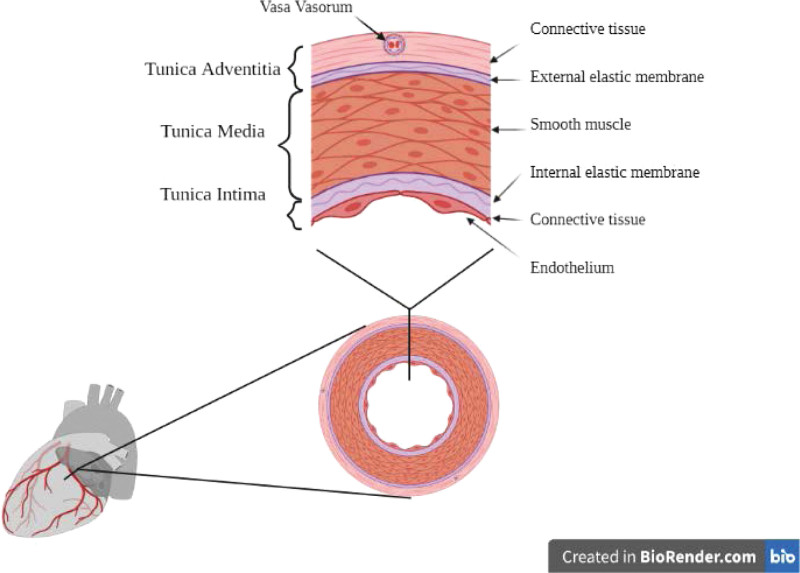
Anatomy of coronary artery wall to help understand the 2 suggested mechanics of spontaneous coronary artery dissection.

The underlying mechanisms of SCAD have baffled the medical community and cardiologists for decades. The disease results from a pathology of the coronary arteries’ endothelium. The coronary arteries are composed of 3 distinct layers, as shown in Figure 1. The innermost layer (tunica intima) is composed of endothelial cells and the internal elastic lamina, a middle layer (tunica media) is composed of smooth muscle cells and the external elastic lamina, and an outermost layer (tunica adventitia) is composed of connective tissue, blood vessels (vasa venorum), and nerves. The underlying pathophysiology of SCAD still remains hypothetical. Regardless of the mechanism of the initial insult, disease occurs due to the separation of the 3 layers of the arterial wall resulting in an IMH and the formation of a false lumen. Two mechanisms have been proposed ^[5,13]^:

The first focuses on an “inside out” mechanism where tears in the tunica intima layer result in an entry point for blood into the intimal space creating a false lumen and an IMH formation.

The second proposes an “outside-in” approach where medial hemorrhage or rupture of the vasa venorum within the tunica adventitia results in hemorrhage into the arterial wall, again, resulting in a blood-filled false lumen and an IMH. It is also plausible that either process can result in SCAD.

## 4. Biorender image

### 4.1. Presentation spectrum

SCAD primarily presents in the nonatherosclerotic ACS setting and must be differentiated from atherosclerotic etiology by coronary angiography or similar coronary imaging modality. The clinical presentation of SCAD has been inextricably related to troponin positive-ACS and has been implicated in 1% to 4% of total ACS occurrences, with SCAD contributing to 40% of MIs in women under 50 years of age. Additionally, roughly half of SCAD occurrences present as ST-elevated-MIs, with more than 75% presenting as a single-vessel disease.^[14]^ In perimenopausal women, there is a strong association with non-coronary fibromuscular dysplasia (FMD). The highest demographic by reported literature for nonatherosclerotic SCAD with associated FMD are postmenopausal Caucasian women (average age of 52.1 + 9.2) presenting with ACS.^[6,16]^

In addition to FMD, additional presentations associated with SCAD such as pregnancy, postpartum period, extreme physical exertion, intense cardiocirculatory stress, extreme emotional stress, migraines, hypertensive crisis, coronary vasospasm, connective tissue/other monogenetic aberrancies, systemic inflammatory conditions such as polyarteritis nodosa and systemic lupus erythematosus (SLE), neurofibromatosis, cocaine, and cabergoline use have been associated with the occurrence.^[14]^ In contrast to prior studies, a US national population-based cohort study involving 66,360 patients found the mean average age of 63.1 + 13.2 years, with only 44.2% in females.^[33]^ Furthermore, depression had the highest concomitance at 5.15% (n = 3415).^[33]^ This cohort study was completed prior to the adoption of the angiographic classification of SCAD that was first proposed by Saw et al in 2014. Therefore, it is possible that the National Inpatient Sample, does not accurately represent the true SCAD patient population.^[61]^

### 4.2. Risk factors

#### 4.2.1. Gender.

SCAD has a 90% predominant occurrence in perimenopausal women with a high percentage (>90%) with concomitant FMD. The data for occurrence in men is much less studied and identifies different risk factors than in women, mostly heavy lifting or isometric exercise (44% occurrence). Men also cite lower levels of traditionally female-associated risk factors for SCAD, such as depression, anxiety, emotional stress, and migraines.^[32]^

It is hypothesized that cardiac physiology may differ between males and females due to the varied responses to androgens. The X-chromosome encodes the expression of androgen receptors. In females, this results in autosomal mosaicism and resultant varied response to androgens including cardiac tissues. Another major physiologic difference is the role of these androgens in relative quantity and flux throughout a woman’s lifetime. Estrogen is a product of testosterone metabolism and varies throughout a woman’s life cycle, specifically with pubertal and menopausal changes. This sequela has an important implication on risk factors intrinsically thought to be tied to SCAD, such as migraine development secondary to autonomic neurovascular dysregulation in postmenopausal women due to estrogen withdrawal. Additionally, the use of hormonal oral contraception and menopause has been thought to indirectly alter cardiac physiology through modification of the negative feedback mechanisms on the hypothalamic-pituitary-gonadal axis with estrogen, predisposing to possible issues surrounding coagulability, atherosclerotic oxidative stress, vasomotor control, and arrhythmogenesis. Finally, women have been found to have structural heart variances compared to the male sex, specifically with regards to coronary tortuosity and coronary microvascular dysfunction secondary to the estrogenic effects on increased metalloprotease activity. Both of which give rise to an increased risk of nonatherosclerotic causes of MI, which is one proposed mechanism of SCAD in women.^[15,23–25]^

Exogenous Hormone: The link between oral contraceptives and hormonal replacement therapy in the incidence of SCAD has limited literature support for significant involvement and has primarily been identified by case reports and one single-center study.^[25,26]^ The pathophysiology of hormonal involvement in SCAD is attributed mainly to the estrogenic effects on increased vascular remodeling secondary to increased metalloproteinase enzymatic activity. Of which, combined oral estrogen and progesterone and estrogen-only oral hormonal therapies have been associated with an increased risk of development of SCAD. Although this link has not been identified as causative for SCAD, it has relegated itself as an associative role in the development of SCAD with concomitant vascular pathologies such as FMD or aneurysmal dilation.^[20–26]^ Additionally, as aforementioned, estrogenic states and hormonal replacement therapy in menopausal women have significant proven effects on coagulability, arrhythmogenesis, vasomotor control, and oxidative stress, all of which can be predisposing factors to SCAD.^[15]^ According to the Saw et al study, combined oral hormonal therapy was not a statistically significant cause of SCAD.^[16]^

Since the report of the first SCAD case, pregnancy was immediately associated with an increased risk of developing ACS secondary to SCAD. The risk factor has been summarized in Table 1.

**Table 1:** Summary of risk factors of SCAD.

Spontaneous coronary artery dissection risk factors	
Gender	90% predominant occurrence in premenopausal woman.^[32]^ With recurrence rate of 44%. ^[32]^
Migraine	According to the US national based cohort 0.8% of patients with SCAD have migraines^[33]^
Arteriopathy and inflammatory diseases	Fibromuscular dysplasia (FMD) 72% to 75.6%.^[16,70]^ Diabetes mellitus, smoking, previous MI, cerebrovascular disease and hypothyroidism contribute to approximately 15%^[16]^ family history of coronary artery disease, hypertension, dyslipidemia contributed to under 40%.^[16]^
Exogenous hormone	Oral contraceptive containing estrogen has been confirmed as a risk factor for SCAD.^[20–26]^ While the data for combined oral hormone therapy remained controversial.^[16]^
Emotional or physical stress	Multiple studies reported the relation between emotional stress and SCAD 26% to 40%.^[16,27]^ Physical stress was found in 16% to 24%^[16,27]^
Pregnancy and postpartum	Scattered represent 15% to 20% of MIs occurring in pregnancy and postpartum^[17]^ with the highest occurrence in; multiparous, women with previous history of fertility treatment and preeclampsia^[28]^ The highest occurrence reported to be within the first month of postpartum. ^[28]^
Connective tissue disorder	Autosomal dominant polycystic kidney disease (ADPKD) 0.09%, Ehlers-Danlos 0.02%^[33]^ with the prevalence highest in type 4,^[34]^ Marfan syndrome 0.02%.^[33]^ Also, Loeys-Dietz syndrome (LDS) has been reported in multiple patients with scant.
Systemic lupus erythematous	Literature suggest SCAD is prevalent in SLE patient approximately 0.2% to 0.42%.^[33,58]^
Corticosteroids	Only 1 case report reported the link between scant and corticosteroids.^[60]^

MI = myocardial infarction, SCAD = spontaneous coronary artery dissection.

Pregnancy and postpartum: Pregnancy and postpartum states have long conferred association with acute SCAD events.^[30,31]^ In 2010, a prospective Mayo clinic study identified that pregnancy-associated SCAD events, primarily manifesting as MIs, were highest in multiparous, high-risk women with the highest occurrence within the first month postpartum. Of particular interest is the lack of extra coronary vessel arteriopathy, including FMD, in these women. However, several other factors were found to contribute to the development of pregnancy-associated SCAD, such as a previous history of fertility treatment and pre-eclampsia.^[28]^ In a nested case-control study, post-SCAD women were found to tolerate lactation and subsequent pregnancies without increased risk for recurrence within a 5-year range; however, this interpretation was limited in validity due to other concomitant risks factors during subsequent pregnancies.^[29]^ This would suggest that pregnancy is an indeterminate risk for the recurrence of SCAD due to the lack of supporting data outside of case series and small prospective studies; however, it would suggest an association between pregnancy and the perineurium state and can be implicated for 15% to 20% of MIs occurring in pregnancy.^[17]^

Connective tissue disorder: Associations have been found between connective tissue disorders such as Ehlers-Danlos (EDS), Marfan, Autosomal Dominant Polycystic Kidney Disease (ADPKD), Loeys-Dietz syndrome (LDS), and Pseudoxanthoma elasticum.^[14]^ One population-based cohort study in 2019 evaluated that among 66,360 patients diagnosed with SCAD in the United States, 60 (0.09%) were associated with ADPKD, 10 (0.02%) had Ehler-Danlos, and 10 (0.02%) had Marfan syndrome (MFS).^[33]^ The low reported values may be attributed to the low prevalence of these connective tissue diseases, and further investigation is required. In numerous case reports, undiagnosed EDS patients were shown to have SCAD as their initial presentation.^[34,42,43]^ Specifically, type IV EDS was found to have a higher predilection for SCAD than other types of EDS; however, it may also be seen in type I and VI.^[34]^ These propose the importance of diagnosing EDS early to affect mortality and outcome. ADPKD is associated with SCAD in several case reports. In 6 case reports reviewed, 4 out of 6 showed dissection of the left anterior descending artery (LAD), with the other 2 in the posterior descending artery and ramus intermedius artery branch.^[44–49]^ A systematic review that included these patients found the median age of diagnosis to be 41 (range: 36–59), with 71.4% of cases occurring in females.^[50]^ ADPKD is due to a defective mutation in polycystic kidney disease-1 and polycystic kidney disease-2 genes, which code for polycystin. Polycystins are found in the vessel walls, specifically in the dense plaques on smooth muscle cell membranes and interlaminar elastic fibers.^[51]^ A study showed that the vascular wall breaks down in the absence of polycystins, leading to rupture and hemorrhage.^[52]^ This mechanism most likely leads to the various vascular abnormalities seen in ADPKD, such as SCAD. Most patients with LDS have a transforming growth factor beta receptor 1 and transforming growth factor beta receptor 2 gene mutation.^[53]^ However, some controversy exists in classifying some patients with the same mutation as MFS due to the Marfan-like phenotype. This may be problematic as the earlier studies reported, that LDS showed more aggressive vascular disease and earlier mortality.^[54]^ However, in a subsequent analysis, there was no difference in the incidence rate of vascular disease and mortality of MFS and LDS patients, so long as they were properly treated.^[55]^ Aneurysms and dissections commonly occur in the aorta (both descending and ascending) and very rarely in the peripheral arteries.^[54]^ In 1 study, analysis of 179 SCAD patients found 17 LDS patients with mutations in SMAD2 (n = 3), SMAD3 (n = 1), TGFB2 (n = 4), TGFB3 (n = 4), transforming growth factor beta receptor 1 (n = 2), and transforming growth factor beta receptor 2 (n = 3), which points to a high frequency of variant among LDS patients. This may suggest that dysregulation of transforming growth factor-beta plays a role in SCAD. Out of this group, 15 out of 17 patients were female, 9 out of 17 occurring in LAD. Age was not specified.^[55]^

Often, patients that presented with SCAD did not have a prior diagnosis of connective tissue disease, albeit some phenotypes were present. Along with the possibility that this patient population is prone to recurrent SCAD and another vascular pathology, genetic testing and or whole-body imaging (computed tomography or magnetic resonance imaging) is recommended in follow-up.^[56,57]^

### 4.3. Migraine

Several studies reported that endothelial dysfunction in migraines plays a role in conditions such as stroke and cervical artery dissection. It is proposed that this correlates to the pathophysiology in SCAD.^[10,11]^ This relation between migraine and SCAD has been studied by the Mayo Clinic research group, who reported primary findings of the predominance of migraines among the studied SCAD population and female sex. They also reported a younger age at the time of SCAD, a higher percentage of depression and post-SCAD chest pain at 1 month, and, finally, higher imaging findings of arterial aneurysms, pseudoaneurysms, and dissections.^[12]^ A US national population-based cohort study involving 66,360 SCAD patients found that 0.8% (545) were associated with migraines.^[33]^

### 4.4. Arteriopathy and inflammatory diseases

The strongest association with arteriopathy and nonatherosclerotic SCAD was in patients identified with concomitant FMD, 72% to 75.6%,^[16,70]^ with the other sizeable predisposing arteriopathy being idiopathic, up to 20.8%. Other minor to relatively noncontributory arteriopathy causes were identified, such as postpartum states, systemic inflammatory conditions, connective tissue disorders, multiparity, multigravidity, and hormonal therapy, all <10.7%. Of the 72% diagnosed with concomitant FMD, 72.7% were found to have renal arterial involvement, followed by iliac arterial involvement at 50.4% and cerebrovasculature involvement at 52.1%.^[16]^ However, in a US national population-based cohort study involving 66,360 patients from 2004 to 2016 comparing SCAD versus non-SCAD ACS cases, the study found that only 0.16% (n = 108) of SCAD cases were associated with FMD. SLE, a specific systemic inflammatory disease, had a higher association rate with SCAD at 0.42% (n = 280).^[33]^

Other inflammatory states or predisposing inflammatory states such as diabetes mellitus, current smoker status, previous MI, cerebrovascular disease, and hypothyroidism all contributed to under 15% of the sample population. In contrast, other notable inflammatory-related factors such as the familial history of coronary artery disease, hypertension, and dyslipidemia all contributed to under 40% of the sample population.^[16]^ Indirect measurement of the SCAD lesions in the Saw et al 2014 study, 67% were found to have diffuse smooth narrowing leading to stenosis with no concomitant atherosclerotic lesions with only 29.1% comprising pathognomonic contrast dye staining lesions, and 3.9% comprising focal or tubular stenosis.^[16]^ This theme that SCAD is more related to FMD (or a precursor of) and less to systemic inflammatory conditions is further corroborated by a multi-cohort review that shows, on average, <5% involvement related to systemic inflammatory states.^[17]^ Postmortem pathological evaluation further differentiated eosinophilic epicardial coronary infiltrative involvement seen in SCAD to be localized to the adventitia and the periadventitial soft tissue from the traditional intimal or medial inflammation seen in that of polyarteritis nodosa or other granulomatous vasculitides.^[17–19]^

Emotional or physical stressors: According to the Saw prospective cohort study in 2014, 40.5% of participants reported a degree of emotional stress precipitating acute events, with 24% of that demographic reporting exercise prior and 12.5% engaging in isometric exercises. Of less statistical significance was the report that other minor activities such as bowel movement straining, severe coughing, and retching/vomiting had even precipitated acute SCAD events. Additional retrospective and prospective studies have identified and suggested in a more recent 2016 study that 26% of participants reported emotional stress, 16% identified physical exercise, further stratifying isometric exercise as only 6% compared to 10% reporting aerobic activity.^[27]^ These studies illustrated that women with concomitant FMD and significant emotional stress were at a higher predisposition to acute SCAD events, whereas men without FMD were more likely to report exercise as the precipitating stressor to acute events.

Systemic Lupus Erythematosus: Association between SLE and SCAD have been found throughout literature. Although some studies suggest between 0.2% and 0.42%,^[33,58]^ the degree of SCAD prevalence in SLE patients is still unclear. In a case series from 2019, 9 SLE-SCAD cases were analyzed. The study found an average of 36.2 years (range 17–53), 7 out of 9 were females, with 8 favorable clinical cases and 1 mortality. Of these, 4 were found in LAD, 4 in left circumflex artery, 2 in obtuse marginal branches, and 1 in right corner artery and posterior descending artery. Two patients presented with more than 1 artery involvement.^[59]^ Due to the disease nature of SLE in conjunction with corticosteroid use being linked to SCAD.^[33,58]^ In this scenario, corticosteroid use may be a confounding factor. However, in 4 out of 9 patients stated above, this episode of SCAD was the initial presentation of SLE. To date, there is only 1 case report that links corticosteroid use with SCAD. A 39-year-old female was diagnosed with SCAD after 3 days of intravenous hydrocortisone 150mg daily and was scheduled to start oral prednisone 60 mg for 7 days. Other possible risk factors were ruled out (i.e., no hormone use, not postpartum, absent pregnancy, negative for FMD and connective tissue disease, negative anti-antinuclear antibodies).^[60]^ This area lacks literature evidence except for a handful of case reports, and more research is needed.

### 4.5. Diagnosis

The gold standard for diagnosis is coronary angiography. SCAD has been classified by 4 major subclassifications, subrogated by the lesion’s type, length, and degree of stenosis.^[17,37–40]^ Type I SCAD presents most similar to a typical arterial intimal dissection with a generation of multiple false vascular lumens stemming from the main vessel. It is important to note that 23% to 40% of the SCAD lesions occur in the LAD and associated proximal vessels.^[16,79]^ This is important as they can be more difficult to manage and maybe more likely to require intervention. One study found Obtuse Marginal artery involvement to be as frequent as LAD.^[79]^ Type 2 SCAD is distinct from that of Type I in that there is no intimal tear but rather an IMH that causes a degree of stenosis within the main vessel. Type 2 lesions are typically long (>20 mm) and are further divided into subtypes A and B, with subtype 2A being defined by an abrupt onset from the regular intramural thickness, and subtype 2B is defined as a continuation to the most distal end of the vasculature.^[17,37–40]^ Type 2 is by far the most common presentation of SCAD, reported to be as high as ⅔ of dissections.^[16,25,79]^ Type 3 is the rarest and is closely related to that atherosclerotic lesions in that it has high degrees of coronary tortuosity, is typically shorter than Type 2 (<20 mm), and must be further differentiated as an IMH versus atherosclerotic lesion. This is usually done by optical coherence tomography (OCT). Type 4 is defined as an IMH leading to complete occlusion of the vessel.^[17,37–40,79]^ One study found mean stenosis to be 78.4% + 18.7% and mean dissection length as 42.7 + 21.3 mm.^[79]^

Intraluminal OCT imaging is perhaps the highest degree of resolution and confirmation when imaging SCAD lesions; however, it comes with a significant risk of iatrogenic lumenal dissection, false cannulation, flow obstruction, and permanent vascular injury, and therefore is reserved for situations in which coronary angiography cannot differentiate between atherosclerotic versus IMH.^[17,41]^ Other imaging modalities for intraluminal analysis include coronary computed tomographic angiography.^[35]^ This modality should be used with a high degree of caution as it has a high false-negative rate for distal lesions and can be inconclusive between true intraluminal hematomas and noncalcified atherosclerotic lesions.^[35]^

For cases that are indeterminate by angiography alone, clinical diagnosis should be considered and appropriately demographic matched. Degree of coronary tortuosity, intraluminal response to nitroglycerin to differentiate coronary vasospasm, and corresponding extra coronary arteriopathy such as FMD should all be considered.^[17,36,39]^

### 4.6. Management

Conservative management: There are ongoing trials investigating the various treatments for SCAD that will be very promising and hopefully provide a more evidenced-based care approach. SCAD management is an area of opportunity due to limited drug or interventional studies. The current BA-SCAD is a prospective randomized, open-label, trial aimed to assess the efficacy of beta-blocker and antiplatelet therapy in patients with SCAD. Alfonso et al study are currently ongoing but it will likely provide evidence regarding the use of pharmacotherapy.^[90]^ According to significant societies, the European Society of Cardiology and the American Heart Association (AHA), conservative management when appropriate is recommended due to the harmful risk of percutaneous intervention (PCI). The European Society of Cardiology released guidelines in 2018 reviewing the treatment options with ultimate recommendations for conservative management in the acute setting. AHA updated guidelines for SCAD management in 2021 recommending further studies to address care for patients with ongoing hemodynamic instability or large areas of myocardial ischemia specifically the LAD or left-main. A trend analysis New Zealand study from 2014 to 2019 observed an increase in SCAD diagnosis, attributed to more patients with non-ST segment myocardial infarction being diagnosed as SCAD. As a result, there was an increase in non-ST segment myocardial infarction-SCAD over the years while ST-elevated-MIs-SCAD stayed relatively the same. Concurrently, an increase in conservative management, as well as a decrease in Major Adverse Cardiovascular Events (MACE) in the 30-day period after SCAD diagnosis, was observed.^[61]^

The push for conservative management stems from 2 main factors: the risk for iatrogenic injuries or intraoperative complications from interventions such as extending the coronary dissection or iatrogenic dissection with a guidewire (wire entry into the false lumen), catheter-induced occlusion of the true lumen (loss of flow after stenting), and hydraulic extension from contrast injection,^[63,66]^ and the ability for spontaneous coronary healing of SCAD lesions over time in conservatively managed patients. Additionally, revascularization at initial onset did not reduce the risk for future PCI/CABG (coronary artery bypass graft) or SCAD events.^[66]^

Iatrogenic catheter-induced coronary artery dissection is reported to be increased in patients with SCAD (prevalence of 3.4%) when compared to non-SCAD patients (prevalence of <0.2%).^[63,64]^ This is due to the underlying arterial fragility.^[62,63]^ In situations of uncertain coronary angiography diagnosis and with a large enough vessel diameter for intracoronary imaging, OCT or intravascular ultrasonography can be performed. However, these modalities pose similar risks and are generally not clinically indicated.^[62]^

Recently, several observational studies had indicated that when a follow-up angiography was performed weeks to months following the initial SCAD event, 70% to 100% of patients showed healing of the lesion.^[16,65–71]^ Evidence shows that spontaneous healing is frequently observed after 1 month.^[16,69–71]^ In a minority of patients, the dissection persisted, and it is unclear why or whether delayed healing will subsequently occur. However, a study of 168 patients initially showed no healing on a follow-up angiography <20 days from the event. Subsequently, elected repeat angiography >26 days (median = 161 days) showed spontaneous angiographic healing in all 79 cases.^[16]^ Another study showed spontaneous healing in 157 of 165 (95.2%) lesions on repeat angiography performed after >30 days (median = 154 days). Another study has reported that out of 94 patients treated conservatively, only 43 of 59 repeat angiography showed spontaneous healing at a median of 2.4 years.^[66]^ There were no baseline angiographic characteristic differences between the lesions that healed and lesions that did not,^[16,70]^ and the characteristics that favor healing remain unclear. Regardless, there is strong enough evidence to support conservative management over invasive interventions when appropriate preferentially.

Of note, early complications of recurrent MI may develop in 4.5% to 10% of conservatively managed patients, primarily from extension of dissection within the first 7 days following an event,^[1,16,72]^ occurring at a mean day of 4 days.^[66]^ These patients may experience recurrent chest pain, ischemia, and SCAD progression on angiography, and the majority require emergency revascularization with either PCI or CABG. For these reasons, in-patient monitoring for an extended period is typically recommended as part of a conservative strategy for SCAD management.^[5,66,73]^

Per AHA, conservative therapy may not be appropriate in high-risk patients, those with ongoing ischemia, left main artery dissection, or hemodynamic instability. It is of consensus that urgent intervention with PCI or CABG should be considered in these patients. The decision should be individualized and based on the expertise of the operator or centers. American Medical Association proposes an algorithm for the management of these cases.^[62]^

Percutaneous coronary intervention: Observational studies have consistently shown that PCI in SCAD leads to clinical complications.^[1,8,16,65–67,72,74,75,91]^ This is predominantly a consequence of arterial fragility from underlying arteriopathy that makes it susceptible to iatrogenic dissections and extension of dissections during PCI. Complications include guidewire entering the false lumen, proximal and or distal dissection propagation during balloon dilation and stent placement, as well as dissection of adjacent branches. Additionally, dissection length can often be extensive, requiring multiple stents to be used in a single vessel, leading to an increased risk of in-stent restenosis or thrombosis. Furthermore, resorption of IMH with healing can result in stent mal-apposition, increasing the risk for stent thrombosis.^[69]^

Frequently, SCAD involves the distal coronary segments, which are too small for stent placement and PCI is unfeasible.^[65,67,68]^ In a study of 189 patients, 95 patients were initially treated with PCI. Of these, technical failure occurred in 30% of patients (under flow-based criteria) and 53% (under residual stenosis-based criteria).^[66]^ Emergency CABG is required in 13%, predominantly due to PCI failure (compared to 2% in the conservative managed population), emergency repeat in 4%, and 1 death. Similar adverse technical outcomes were seen regardless of flow parameters (preserved vs reduced flow) at baseline. Of note, patients in the PCI group were found to have higher levels of vessel occlusion (48% vs 19%) and mean lesion stenosis (90% vs 75%). Interestingly, no significant difference in mortality rates was found at follow-up (median = 2.3 years).^[66]^

In a 2015 study, out of 134 patients, 51 underwent PCI, and 5 underwent emergency CABG as initial management. Of these, technical failure was seen in 27%, and emergency CABG due to PCI failure occurred in 5.8% (3 patients). One death secondary to retrograde aortic dissection after PCI and 1 death secondary to cardiogenic shock from initial CABG treatment occurred. Five revascularized patients experienced MI, primarily due to stent thrombosis. MACE rate was 16% in revascularized patients (3.8% in conservatively managed). Again, no significant differences were observed in long-term outcomes.^[72]^ In another study, out of 168 SCAD patients, 28 underwent PCI as initial management, with 5 PCI after failing conservative management. The technical failure occurred in 36%, stent thrombosis in 6%, and emergency CABG due to PCI failure in 12%. Of note, among successful PCIs, procedure extension of dissection occurred in 57%. Merely 30% of PCI procedures had successful and durable results without any complications.^[16]^ As noted prior, revascularization at initial onset was not associated with reduced risk for future PCI/CABG or SCAD events.^[66]^

In conclusion, PCI should be reserved for the high-risk population of SCAD patients that fail conservative management or develop early complications. As noted above, early complications of recurrent MI may develop in 4.5% to 10% of conservatively managed patients, primarily from extension of dissection within the first 7 days following an event,^[1,16,72]^ occurring at a mean day of 4 days.^[66]^ Patients without ongoing signs of ischemia should be closely monitored for an extended period, and decisions for revascularization should be individualized to each case.

Coronary artery bypass graft: CABG in SCAD is generally preserved as a bail-out strategy either for PCI technical failure (e.g., persistent ischemia, failure to pass the wire) or due to the significance and extent of the dissection where conservative management and PCI would be contraindicated.^[76]^ There is limited literature involving CABG in SCAD and is composed primarily of case reports and series.^[1,16,66,67,77]^ Indications for revascularization may include left main coronary artery or severe proximal 2-vessel dissection, active ischemia, hemodynamic instability, cardiogenic shock, or ventricular arrhythmias.^[5,62,77]^ However, judgment should be exercised depending on the clinician’s expertise.

Of the 6 patients who received CABG as initial treatment, in-hospital survival was 100%. Twenty patients with SCAD underwent CABG at some point during the initial hospital course. Two of the thirty-four intended bypass targets could not be bypassed due to the extent of dissection. These were in secondary vessels.^[66]^ Of note, only 5 of 16 grafts were patent on follow-up angiography (follow-up was performed on 11 out of 20 patients).^[66]^ Another study reported, >70% of CABG patients had occluded bypass vessels at follow-up.^[16]^ This is due to spontaneous arterial healing resulting in eventual graft failure from the competitive flow. CABG also does not provide long-term protection against recurrent SCAD as the vast majority of recurrences are de novo (i.e., occurring in a different coronary segment)^[1,16]^

In conclusion, conservative therapy is generally preferred in the absence of ongoing ischemia or clinically stable patients. Although CABG may be necessary for specific clinical circumstances listed above, the long-term outcome remains poor compared to the favorable outcome observed in conservative management.^[66,74]^

Medical management: Conservative medical management consists of managing chronic chest pain, prevention of SCAD recurrence, reproductive counseling, assessment for and managing extra coronary vascular abnormalities, and finally, improvement of quality of life. Guidelines for medical SCAD management are based solely on registry data and expert consensus due to a lack of prospective trials.

Anticoagulation: Aligning with MI treatment guidelines,^[80,81]^ anticoagulation and dual antiplatelet therapy are often initiated before the diagnosis is made. Given the unapproved benefit and most appropriate duration of anticoagulation in SCAD treatment along with the hypothetical risk of dissection extension due to worsening of intramural bleeding, and the association of thrombolysis treatment with clinical deterioration in patients, expert consensus is that these medications should be discontinued after the diagnosis of SCAD has been confirmed on angiography.^[62,76]^

Antiplatelet Therapy: Same as anticoagulants, the lake of data for dual antiplatelets, most of the recommendations are derived from the general expert consent who apparently agrees on administrating dual platelets in the acute phase for 1 year with different duration based on the patient’s underlying cause for SCAD.^[80,81]^ For example, it has been recommended that patients with SCAD with FMD will benefit from long-term treatment, while this is not the case in premenopausal women with menorrhagia.^[82]^

Beta-Blockers, angiotensin receptor blockers and angiotensin-converting enzyme inhibitors: The recommendation for these medications remain persistent with the current MI and heart failure treatment guideline.^[81]^ Also, Beta-blockers might hold the extra benefit of preventing the recurrence of SCAD. It has been reported that the use of beta-blockers resulted in a 64% decrease in the recurrence of SCAD over a median of 3.1 years.^[79]^

Statin: Since SCAD is not mediated by atherosclerotic plaque rupture, with a lack of data supporting routine administration of statins after MI due to SCAD data are lacking to support the routine use of statins after MI due to SCAD, the use of statins may be limited to patients who otherwise meet major criteria for treatment guidelines of hyperlipidemia.^[1,79]^

Antianginal Therapy: Chest pain in patients who suffer from SCAD is a frequent reason for hospital readmissions. SCAD is responsible for about 20% of readmissions within 30 days after acute MI.^[83]^ It is essential to know that chest pain may continue for several months after SCAD with negative evaluations of ischemia.^[84,85]^ In patients who continue to have atypical chest pain with standard ischemia workup, It is crucial to consider coronary vasospasm, endothelial dysfunction, microvascular disease, catamenial chest pain, and noncardiac chest pain. Medical management includes calcium-channel blockers, nitrates, and ranolazine.^[62]^

Medical management of Hypertension: In a multivariate Cox regression analysis, 1 study found a 2-fold increased risk of recurrent SCAD in hypertensive patients.^[79]^ Another crucial finding was that an underlying history of hypertension predicted recurrent SCAD. It is crucial to remember that hypertension increases the risk of local fatigue and endothelial damage by inducing arterial remodeling, including the proliferation of vascular smooth muscle cells and breakdown of medial elastin, and by increasing the circumferential arterial wall stress.^[79]^ These acute and chronic arterial changes can empirically increase the risk of arterial dissection Hypertension should be treated adequately to prevent the recurrence of SCAD.^[78,79]^ Beta-blockers are the preferred antihypertensive class in patients with SCAD due to a multivariate Cox regression analysis reporting that beta-blocker use was associated with a reduced risk of recurrent SCAD (heart rate: 0.36).^[79]^

Prevention of SCAD recurrence: The recurrence rate of SCAD has been reported in several studies with variable follow-up times ranging from 2.3 to 3.9 years.^[1,66,79]^ The reported rates of recurrent SCAD varied from 10% to 17%. Of these studies, 2 have reported Kaplan–Meier estimations of recurrent SCAD at 5 and 10 years, reported at 27% and 29.4%, respectively.^[1,66]^ The majority of cases of recurrent SCAD occur in de-novo coronary arteries and in female patients. Recurrent rates of SCAD were similar in patients who were treated conservatively or with revascularization for their initial event. Multiple risk factors for recurrent SCAD have been evaluated, although large randomized clinical trials for these variables are lacking, and the data is contradictory. Underlying arteriopathies, including non-coronary FMD, have been reported to associate with SCAD in multiple studies.^[1,6]^ In general, this is thought to be secondary to the weakening of the coronary arteries, although no histological proof exists to establish a causal relationship. Furthermore, coronary tortuosity was evaluated as an angiographic predictor of recurrent SCAD in a study by Eleid et al.^[39]^ Severe coronary tortuosity was thought to have a borderline association with recurrent SCAD. In addition, markers of tortuosity were associated with extra coronary vasculopathy, including FMD.

Another significant independent predictor for recurrent SCAD is systemic hypertension^[79]^ due to the increased shear stress on arterial walls. Consequently, factors that reduce blood pressure, such as antihypertensive medications and exercise, and those that may increase it, such as physical (specifically heavy isometric exertion) and emotional stressors, can potentially play a role in recurrent SCAD. Of the several pharmacologic agents that have been used in SCAD patients (including aspirin, beta-blockers, calcium-channel blockers, ACE-inhibitors, and statins), only beta-blockers have been shown to reduce the risk of recurrent SCAD.^[79]^ In 1 study,^[1]^ statin use was higher in the SCAD recurrence group; however, the data analysis was limited. Further studies, such as the SAFER-SCAD trial, regarding the role of statins and ACE-inhibitors in preventing recurrent SCAD, are underway. Lastly, a dedicated cardiac rehabilitation program for SCAD patients was developed and studied by Chou et al,^[85]^ which used a multidisciplinary approach to address both the physical and psychosocial aspects of SCAD events. The program was shown to be beneficial in improving symptoms, exercise capacity, and psychosocial well-being. However, it did not demonstrate the ability to reduce the recurrent rate of SCAD.

Management of quality of life: SCAD patients have a big impact on their quality of life due to migraine headaches, depression, anxiety, and post-traumatic stress disorder PTSD which are common findings post SCAD.^[12,86]^ That being said, early detection and treatment of these complications and referral for these conditions are recommended.^[62,87]^ Also, cardiac rehabilitation is recommended and proven to be safe and might improve emotional well-being, and decrease depression, stress, and chest pain.^[85,88,89]^

Prognosis: Overall, all-cause mortality remained unchanged between revascularized patients and conservatively managed patients. However, MACE was higher in revascularized patients.^[72]^ One study reports a 6-year survival rate of 94% in SCAD patients, while another study reports a ten-year survival rate of 93%.^[1,72]^ Short-term hospital courses in conservatively managed patients had a 4.6% recurrent MI rate and 4.3% unplanned revascularization with 100% early survival at discharge. However, at long-term follow-up (median = 3.1 years), the recurrent MI event rate was 16.8%, with 61.9% due to recurrent SCAD. Recurrent SCAD was seen in 10.4% of all SCAD patients.^[79]^

## 5. Conclusion

High clinical suspicion for SCAD is necessary during the evaluation of ACS. SCAD disproportionately affects women with 90% of cases being in females. Additionally, women are younger than traditional ages for atherosclerotic-related MIs. Coronary angiography is the diagnostic test of choice. There are no randomized controlled studies that validate pharmacotherapy or interventions for treatment. The European Society of Cardiology and the AHA updated recommendations strongly favors conservative treatment for the majority of cases. Consideration for evaluation of other risk factors and comorbid conditions such as FMD should be considered on a case-by-case basis. SCAD has a higher rate of reoccurrence and is associated with worse outcomes than traditional atherosclerotic disease. Clinical trials and research is needed to provide evidence-based practice guidelines.

## Acknowledgments

The authors thank Librarian Janet Hobbs for her great help getting in getting access to all papers reviewed.

## Author contributions

**Conceptualization:** Bashar Khiatah, Sam Jazayeri.

**Data curation:** Bashar Khiatah.

**Formal analysis:** Sam Jazayeri.

**Validation:** Amanda Frugoli.

**Visualization:** Amanda Frugoli.

**Writing – original draft:** Bashar Khiatah, Naofumi Yamamoto, Tristen Burt.

**Writing – review & editing:** Sam Jazayeri, Amanda Frugoli, Dennis L Brooks.
